# Microstructural Stability of IN625 Reinforced by the Addition of TiC Produced by Laser Powder Bed Fusion after Prolonged Thermal Exposure

**DOI:** 10.3390/ma17184532

**Published:** 2024-09-14

**Authors:** Serena Lerda, Giulio Marchese, Emilio Bassini, Mariangela Lombardi, Daniele Ugues, Paolo Fino, Sara Biamino

**Affiliations:** 1 DISAT-Department of Applied Science and Technology, Politecnico di Torino, Corso Duca degli Abruzzi 24, 10129 Torino, Italy; emilio.bassini@polito.it (E.B.); mariangela.lombardi@polito.it (M.L.); daniele.ugues@polito.it (D.U.); paolo.fino@polito.it (P.F.); sara.biamino@polito.it (S.B.); 2 IAM@PoliTo-Interdepartmental Center of Integrated Additive Manufacturing, Politecnico di Torino, Corso Castelfidardo 51, 10129 Torino, Italy; 3 INSTM-Consorzio Interuniversitario Nazionale per la Scienza e Tecnologia dei Materiali, Via G. Giusti 9, 50121 Firenze, Italy

**Keywords:** Ni-based superalloys, Inconel 625, laser powder bed fusion, composite, heat treatments, titanium carbides

## Abstract

This paper deals with the development and characterization of an Inconel 625 (IN625) reinforced with 2 wt.% of sub-micrometrical TiC particles produced by the laser powder bed fusion (LPBF) process. IN625 and IN625 2 wt.% TiC microstructural evolution was evaluated in the as-built, solution-annealed (2 h at 1150 °C), and prolonged heat-treated (2 h at 1150 °C + 100 h at 1000 °C) conditions. The IN625 and IN625 + TiC samples were successfully produced with low residual porosity (<0.15%). In the as-built conditions, both materials developed mainly columnar grains elongated to the building direction with melt pools, fine dendric structures, and small fractions of recrystallized grains. Some TiC segregations were observed in the composite, preferentially located at the melt pool boundaries. The heat treatments led to a different microstructural evolution between the base alloy and the composite. After solution annealing, the IN625 alloy was subjected to full recrystallization with a drastic reduction in hardness. Afterward, the prolonged thermal exposures for 100 h at 1000 °C provoked the formation of carbides, increasing the hardness. On the contrary, the composite retained the as-built microstructure with columnar grains in the solution-annealed and prolonged heat-treated conditions, revealing a limited formation and growth of carbides, thus resulting in a reduced hardness variation. The addition of TiC inside the IN625 enhanced the microstructural stability of the composite, preventing the recrystallization and the growth of phases occurring under prolonged thermal exposures. The current study therefore reported the effect of TiC particles on the microstructural stabilization of LPBFed IN625, with a peculiar focus on the prolonged thermal exposure at 1000 °C.

## 1. Introduction

Laser powder bed fusion (LPBF) is one of the most promising additive manufacturing (AM) processes. Starting from metallic powder, a laser beam selectively melts the powder layer by layer in a chamber protected by an inert gas atmosphere. The LPBF process allows the production of near-net-shape products in a single step, becoming especially attractive for the manufacture of complex-shaped components made of hard machinable materials [1,2,3].

Ni-based superalloys represent one of the most critical categories since, during subtractive operation, the heat generated by the combination of high hardness and low thermal conductivity considerably reduces the life of cutting tools [4]. For all these reasons, nowadays there is solid literature knowledge about the processability of several Ni-based superalloys through the LPBF process, chiefly focusing on the process parameter optimization and investigation of heat treatments to improve their mechanical performances [5,6,7,8].

Moreover, in recent years, there has been a growing interest in the production of metal matrix composites (MMCs) through the LPBF process in order to produce complex-shaped MMC components in a single step, thus overcoming the issue related to their reduced machinability derived from the addition of ceramic particles [9,10].

Among the MMCs, Ni-based composites were investigated to increase mechanical performances and thermal stability, thus enabling the application in more severe conditions [11,12]. Due to its excellent weldability compared to other Ni-based superalloys, Inconel 625 (IN625) has been considered and studied as a successful metal matrix for composite production [13]. IN625 is classified as a solution-strengthened Ni-Cr-based superalloy offering excellent oxidation and corrosion behavior up to around 1000 °C [14,15]. Moreover, the reaction between Ni and Nb can form a metastable coherent body-centered tetragonal γ′′ (Ni_3_Nb) phase, which enhances the mechanical performances up to 650 °C. However, after a prolonged thermal exposure between 650 °C and 900 °C, γ′′ phase transformed into the stable incoherent and brittle orthorhombic δ (Ni_3_Nb) phase with a severe reduction in the ductility of the alloy. Furthermore, the presence of other alloying elements leads to the formation of different types of carbides, such as (Nb, Ti-rich) MC, (Mo-rich) M_6_C, and (Cr-rich) M_23_C_6_ carbides. MC, M_6_C, and M_23_C_6_ carbides can act as a strengthening phase in the alloy, increasing the mechanical performance [16,17,18]. IN625 was studied with the addition of different ceramic particles (SiC, WC, TiB_2_, and YSZ [13,19,20,21]) for MMC production; however, the most promising one was identified as TiC. The addition of TiC guaranteed good chemical affinity with IN625 and great hardness increment. Moreover, TiC has higher thermal conductivity and laser absorptivity than IN625, thus increasing the temperature inside the melt pool during the melting [13,22,23].

According to the literature, the laser powder bed fused (LPBFed) IN625 + TiC composite was investigated using different sizes and weight percentages (wt.%) of TiC particles. Chen et al. [24] investigated the addition of nanometric TiC (mean size 40 nm). A 4 wt.% IN625 + TiC nanocomposite was produced at different laser energies and deeply characterized in the as-built condition. The samples produced at higher laser energy guaranteed the realization of nearly fully dense samples with the strongest increase in mechanical properties compared to the IN625. Furthermore, this condition reported a clear TiC refining effect on the IN625 dendritic structures not detected using lower energy to process the material. Chen et al. [25], in another research work, evaluated the oxidation behavior of the 4 wt.% IN625 + TiC nanocomposite at high temperatures. In the heat-treated condition, the TiC decomposition towards TiO_2_ reduced the Fe diffusion in the composite, leading to the formation of a dense Cr_2_O_3_ protective layer. Two former works of our research group [26,27] discussed the IN625 reinforced with micrometrical TiC (1–5 µm) and sub-micrometrical TiC (mean size 200 nm). The use of micrometrical TiC [26] allowed to understand the potentiality of the added particles to improve the microstructure stability of the material, but their size limited the dissolution of TiC and highly reduced the flowability, making it impossible to further increase their content above (1 wt.%). Subsequently, the reduction in flowability, and consequently, processability, was solved by using sub-micrometric TiC [27], and the resulting material presented higher hardness and tensile strengths compared to the IN625 alloy in the as-built and two different heat-treated conditions. However, to the authors’ knowledge, there are no studies that paid attention to the microstructure evolution of the LPBFed IN625 + TiC composite under prolonged thermal exposures, which can influence the mechanical performance of the material under service at high temperatures. It is therefore crucial to perform deeper investigations to understand the microstructural stability of the IN625 + TiC composite under prolonged thermal exposures. This paper is focused on the microstructure and hardness evolution of the LPBFed IN625 + TiC material under prolonged thermal exposures compared to the IN625 alloy.

So, in the current study, an IN625 alloy with the addition of 2 wt.% sub-micrometric TiC composite was successfully produced and characterized. In particular, the microstructural features and the hardness values were compared to the IN625 base alloy in as-built and heat-treated conditions. The effect of TiC particles in preventing recrystallization and grain growth is studied for prolonged thermal exposures, showing delayed kinetic formation and growth of carbides compared to the prolonged heat-treated IN625 alloy.

## 2. Materials and Methods

### 2.1. Powder Characterization

IN625 gas-atomized powder was used as the metal matrix and reference alloy in sample production. IN625 powder was purchased by EOS GmbH (Krailling, Munich, Germany) with a stated D_50_ of around 35 μm. The chemical composition and morphology of IN625 powder were analyzed using a scanning electron microscope (SEM, Phenom XL, Phenom-World, Eindhoven, The Netherlands) with an energy dispersive spectroscopy (EDS) system. Moreover, a LECO detector (LECO, CS 744 LECO-analyzer, Leco, St. Joseph, MI, USA) was used to determine the C content. The evaluated chemical composition (Table 1) fell within the range reported in the UNS N06625 standard ASTM B446-19 [28].

**Figure 1:**
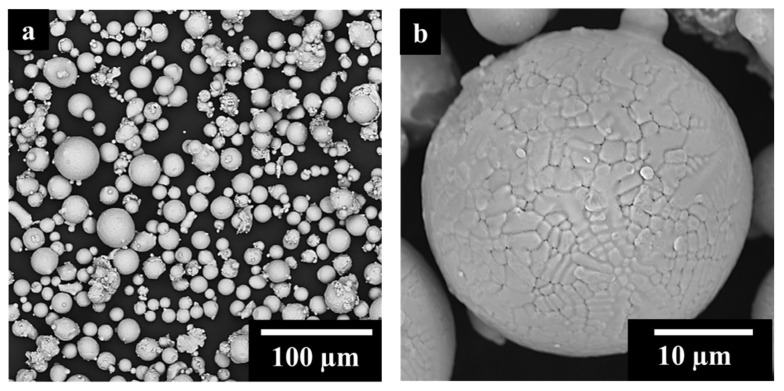
IN625 particles low magnification (**a**) and high magnification (**b**) SEM images.

Figure 1a shows almost spherical IN625 powder particles with some sporadic satellites. At high magnification (Figure 1b), IN625 particles revealed a dendritic structure on the surface generated by the elevated cooling rates occurring during the gas-atomization process.

**Figure 2:**
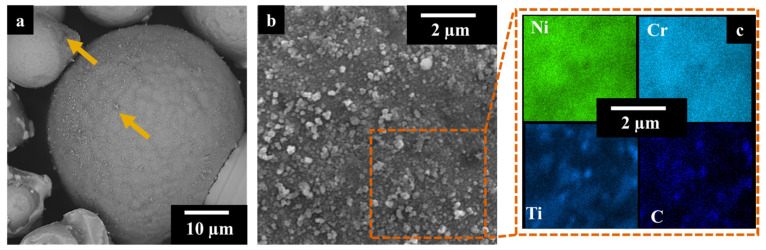
SEM images of IN625 + TiC powder after mixing (**a**), the yellow arrows pointed TiC agglomeration; high magnification SEM image of IN625 + TiC after mixing (**b**) with EDS maps on the IN625 decorated surface by TiC (**c**).

As ceramic reinforcement, the TiC particles used were purchased by Sigma-Aldrich (Merck KGaA, Darmstadt, Germany) with a given average diameter of 200 nm. The IN625 added by 2 wt.% of TiC powder was mechanically dry-mixed in jars for 24 h, applying a speed rate of 60 rpm. The mixing was performed at room temperature, and no inert atmosphere was implemented. Figure 2a displays the presence of some TiC agglomeration (yellow arrows) due to the strong Van der Waals forces acting on the sub-micrometrical particles. The IN625 powder surfaces were decorated by TiC particles, as pointed out by the SEM images and EDS analysis in Figure 2b,c.

Before the LPBF process, IN625 and IN625 2 wt.% TiC (hereafter called IN625 + TiC) powders were sieved below 50 μm and tested with the Carney flowmeter funnel following the ASTM B964-23 [29]. The results are reported in Table 2. The addition of 2 wt.% TiC particles (sub-micrometrical) slightly worsened the flowability behavior compared to IN625 1 wt.% TiC (sub-micrometrical) powders. Both 1 wt.% and 2 wt.% showed better flowability than the IN625 1 wt.% TiC micrometric. Finally, the addition of 2 wt.% of sub-micrometrical TiC represented a successful improvement compared to the micrometrical 2 wt.% composite powders, which could not flow through the Carney flowmeter. It should be noted that the Carney flowmeter funnel was used since the powder did not flow in the Hall flowmeter funnel.

### 2.2. Building Process

The IN625 and IN625 + TiC powders were processed through an MLab Cusing R Machine (Concept Laser GmbH, Lichtenfels, Germany) exploiting a maximum 100 W power fiber laser with a spot size of around 50 μm. In this study, the volumetric energy density (VED) was set at 99 J/mm^3^ with 70 W as laser power, 900 mm/s as scanning speed, 0.04 mm as hatching distance, and 0.02 mm as layer thickness. Cubes with dimensions 15 mm × 15 mm × 10 mm were produced using a scanning strategy of 5 mm stripes and 67° rotation between each layer. No pre-heating temperature was applied to the building platform.

### 2.3. Heat Treatments

A two-step heat treatment was performed on as-built (AB) IN625 and IN625 + TiC to investigate the microstructural evolution under prolonged heat treatment. The microstructural characterization was performed after each heat-treated step. The first step consisted of a solution-annealing treatment (SOL) for 2 h at 1150 °C, while the second one was performed as a prolonged thermal exposure, hereafter abbreviated as aging treatment (SOL + AG) for 100 h at 1000 °C to investigate the microstructure and hardness stability [30]. All the heat treatment steps were followed by water quenching to prevent any microstructural changes during the cooling step. All the heat treatments were performed in a horizontal tube furnace (RHTC 80-710/15, Nabertherm GmbH, Lilienthal, Germany) with a heating rate of 10 °C/min.

### 2.4. Microstructure and Hardness Characterizations

The IN625 and IN625 + TiC samples in AB, SOL, and SOL + AG conditions were cut along the building direction (z), grounded with SiC papers, and polished with 1 μm diamond suspension. Residual porosity was evaluated using a light optical microscope (LOM, Leica DMI5000M, Wetzlar, Germany). Fifty images at 500× magnification were analyzed through ImageJ software (version: v1.53k, National Institutes of Health, Bethesda, MD, USA) for AB IN625 and IN625 + TiC samples. Furthermore, for the AB IN625 + TiC sample, TiC segregations were identified using EDS analysis and then evaluated in terms of area percentage distribution and dimensions using LOM images. The analyses were performed on 20 images at 500× magnification and then post-processed with ImageJ software.

Microstructural characterization was performed through LOM, SEM, and focused ion beam scanning electron microscope (FIB-SEM) (TESCAN S9000G, Tescan Company, Brno, Czech Republic) equipped with an electron backscattered diffraction (EBSD) detector. For the EBSD analysis, the samples underwent an additional final polishing step with colloidal silica suspension (0.04 µm). The EBSD analysis was performed by scanning samples at 20 kV and 10 nA using a step size of around 2 µm with a tilting angle of 70°. The recrystallization was investigated by means of the grain orientation spread (GOS) maps, where the grains with GOS ≤ 2° were considered recrystallized [31,32,33]. For microstructural feature observations by means of LOM and SEM, the samples were electrochemically etched with a hydrochloric acid and methanol solution (1:1) at 4.5 V for 3 to 5 s. The carbides fraction and dimension were evaluated by post-processing 5 SEM images at 5000× for each condition (SOL and SOL + AG IN625 and IN625 + TiC) with ImageJ software. The high contrast of back-scattered SEM images makes it possible to separate the carbides from the matrix, thus allowing their evaluations.

Finally, the Vicker microhardness (HV) test was carried out on AB, SOL, and SOL + AG IN625 and IN625 + TiC samples. The microhardness test was conducted with a DHV-1000 digital microdurometer (HUATEC Group Corporation, Beijing, China) using 100 gf and 15 s dwell following the ASTM E92-23 [34]. Five indentations were performed 1.5 mm far from the edge, and among them were performed on two samples for each condition.

## 3. Result and Discussion

### 3.1. Porosity and TiC Homogenization

**Figure 3:**
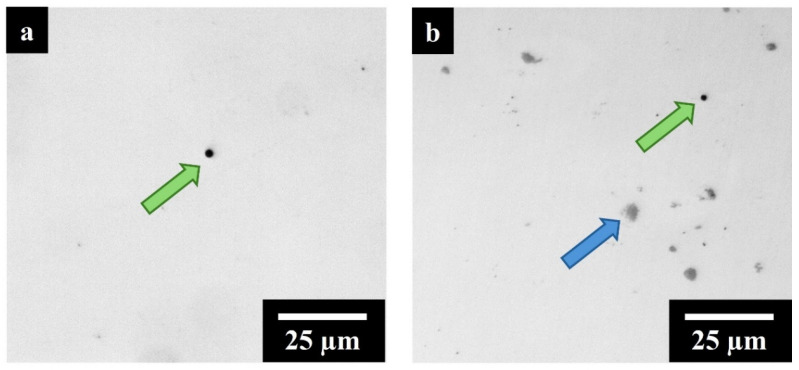
(**a**) LOM AB IN625 image (green arrows pointed out voids) (**b**) LOM AB IN625 + TiC image (green and blue arrows pointed respectively voids and TiC clusters).

Figure 3a,b displays a LOM image, respectively, of AB IN625 and IN625 + TiC samples used for porosity evaluation.

The image analyses revealed a low residual spherical porosity (green arrow) with a maximum calculated value of 0.15% for both the alloy and the IN625 + TiC. This outcome highlighted the negligible reduction in the composite processability with the addition of 2 wt.% of sub-micrometrical TiC particles, showing residual porosity values compatible with the base alloy IN625 and IN625 1 wt.% TiC composite (maximum 0.15% residual porosity) [27]. The high densification levels can be attributed to the higher laser absorptivity and thermal conductivity of TiC than IN625, leading to an increase in the temperature in the melt pools during the melting and consequently, providing a faster heat distribution [22,35]. However, a VED of 99 J/mm^3^ hindered the full dissolution of TiC particles in the melted IN625 alloy, leading to the presence of TiC clusters, as shown by the blue arrow in Figure 3b. The area percentage distribution of TiC was 0.6% ± 0.2% with dimensions ranging from 0.2 µm to 10 µm. Further investigation was required to investigate the cluster formation process during the LPBF melting.

### 3.2. Microstructural Features Evaluation of AB Condition

**Figure 4:**
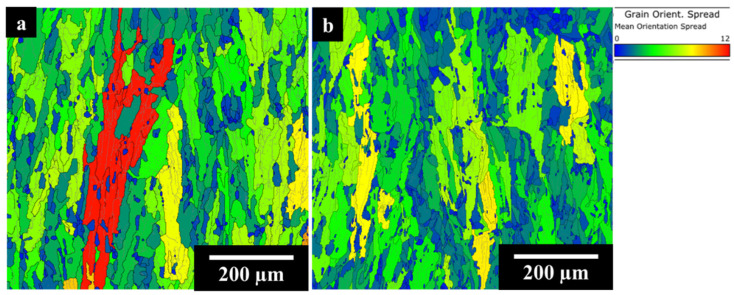
EBSD GOS maps of IN625 (**a**) and (**b**) IN625+ TiC.

The EBSD GOS maps of the AB IN625 and AB IN625 + TiC samples are displayed in Figure 4a and Figure 4b, respectively.

The IN625 and IN625 + TiC revealed similar grain structures with columnar grains elongated to the building direction following the heat flow dissipation during the LPBF process. IN625 displayed high grain distortion with a fraction of recrystallized grains (GOS < 2°) of 20%. On the other hand, the composite showed less tension in grain structure coupled with a 28% fraction of recrystallized grains (and consequently an increment of 40% compared to the IN625 alloy). The high cooling rates of the LPBF process increase the residual stresses on the material, thus generating distorted grains, while the remelting induced by the laser beam during the layer-by-layer process tends to promote the formation of small recrystallized grains. The role of ceramic particles could be useful in increasing the number of the small recrystallized grains, acting as a nucleation site [23,24].

**Figure 5:**
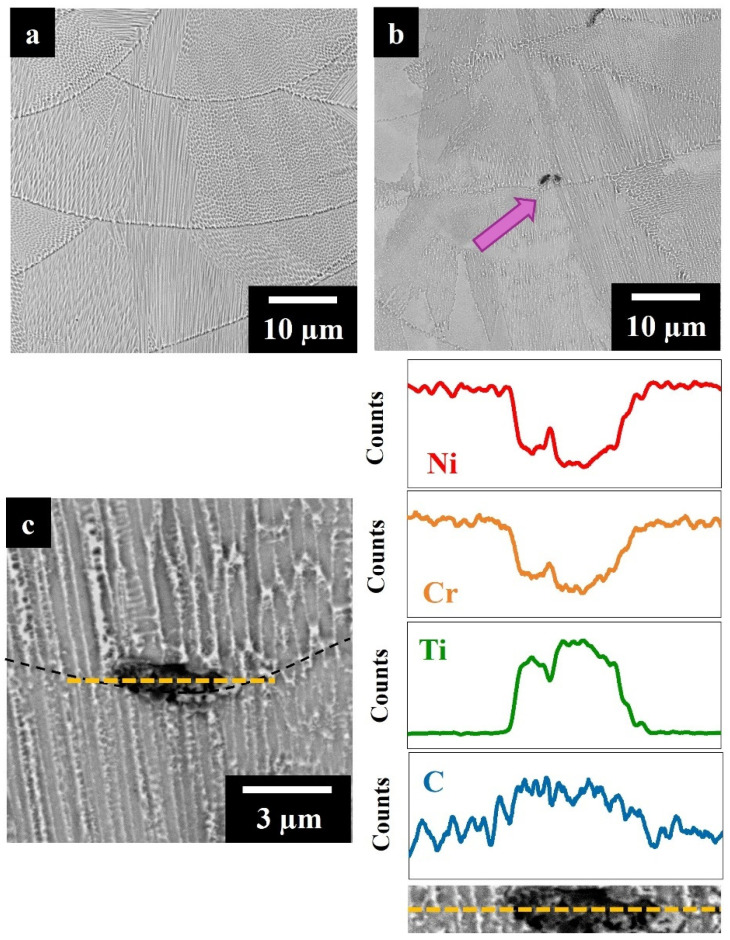
SEM images of (**a**) IN625 and (**b**) IN625 + TiC (the pink arrow pointed out a TiC segregation). (**c**) SEM image focusing on TiC segregation with line EDS analysis.

Figure 5a,b reveals melt pools and a fine dendritic microstructure in both AB IN625 and IN625 + TiC. Both the microstructural features could be associated with the LPBF process. The laser scan led to the presence of melt pools, while the rapid cooling undergone by the molten metal promoted the segregation of alloying elements and the formation of fine dendritic structures [36,37,38]. In addition, the composite showed the presence of TiC segregations decorating the melt pool contours (as indicated by the pink arrow in Figure 5b). Figure 5c at higher magnification showed the segregations where the SEM + EDS analysis confirms the identification of TiC particles. The presence of segregated TiC zones can be correlated with the combined effect of two main phenomena. The strong cohesive Wan der Walls forces drove TiC sub-micrometrical particles to assemble in clusters. When the laser beam melts the powder, a part of the cluster of TiC could remain unmelted in the liquid inside the melt pool, and subsequently, the Marangoni flow pushes them toward the melt pool contours [27,39]. However, a part of the TiC is melted by the laser beam, and the Ti is dissolved into the gamma matrix, while the C is enriched in the interdendritic and grain boundary areas, promoting the formation of new carbides during cooling [27].

### 3.3. Microstructural Features Evaluation of Heat-Treated Conditions

**Figure 6:**
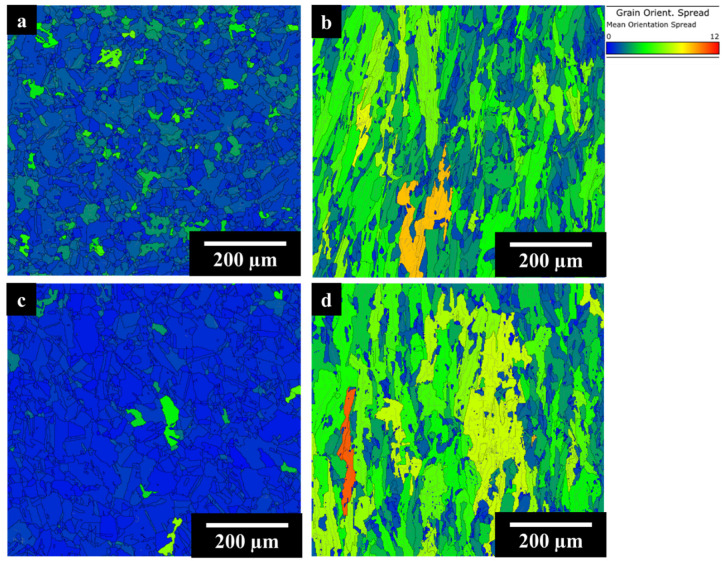
EBSD GOS maps of SOL IN625 (**a**) and IN625 + TiC (**b**) and SOL + AG IN625 (**c**) and IN625 + TiC (**d**).

Figure 6 shows the EBSD GOS maps respectively for IN625, IN625 + TiC in SOL (a and b) and SOL + AG (c and d) conditions.

SOL IN625 revealed 90% of recrystallized grains with the formation of equiaxial grain structure with grain sizes ranging from 8 µm to 130 µm. The solubilization and recrystallization strongly reduce the residual stress inside the grains. Conversely, the composite still exhibited elongated grains as in the AB condition, with dimensions between 6 µm and 400 µm and a higher concentration of deformed grains (higher GOS values) compared to the SOL IN625 alloy. In this condition, the fraction of recrystallized grains was calculated as 26%, overall almost equal to the AB condition of 28%, thus confirming that the addition of TiC particles to the IN625 matrix allowed the stabilization of the columnar grains due to their pinning effect on the grain boundaries.

After prolonged soaking for 100 h at 1000 °C, SOL + AG IN625 showed equiaxed grains with dimensions from 8 µm to 140 µm, similar to the SOL conditions (8 µm to 130 µm), revealing that the thermal exposures did not provoke significant grain growth and the fraction of recrystallization grains resulted in 95%, slightly higher than the SOL condition (90%). This scenario is fully in line with the work of Suave et al. [16], which reported negligible grain modification and coarsening during 100 h aging heat treatment for the traditional processed IN625 alloy. On the other hand, the SOL + AG IN625 + TiC still retained the columnar grains with sizes between 8 µm and 480 µm, slightly above the SOL IN625 + TiC (8 µm to 400 µm). The recrystallized grain fraction was found almost unaltered compared to the SOL sample (26%), with a fraction of recrystallized grains of 25%. The GOS outcome for SOL + AG samples confirmed the stability of the composite microstructure after prolonged thermal exposures for 100 h at 1000 °C.

**Figure 7:**
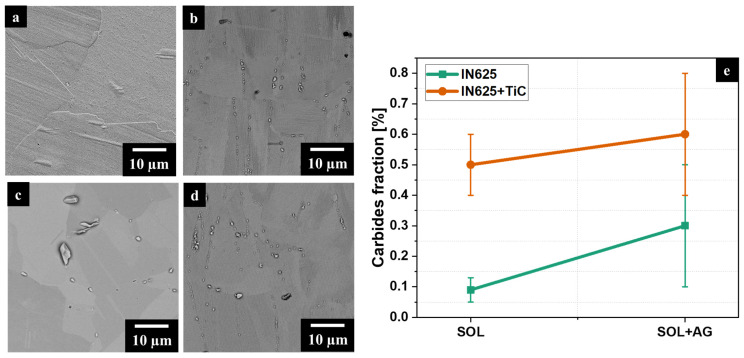
SEM images of SOL IN625 (**a**) and IN625 + TiC (**b**) and SOL + AG IN625 (**c**) and IN625 + TiC (**d**). Carbides fraction evolution between SOL and SOL + AG condition for IN625 and IN625 + TiC (**e**).

SOL IN625 (Figure 7a) and SOL IN625 + TiC (Figure 7b) conditions displayed great differences in terms of microstructural features. SOL IN625 showed recrystallization with a total dissolution of the melt pools and dendritic structures. On the contrary, the SOL IN625 + TiC underwent a partial dissolution of the dendritic microstructure associated with carbide precipitation inside the grains and along the grain boundaries. The intergranular carbides presented a larger size than the intragranular carbides. Furthermore, the prolonged thermal exposures in SOL + AG IN625 (Figure 7c) and SOL + AG IN625 + TiC (Figure 7d) induced the growth of the carbides more pronounced for the IN625 than IN625 + TiC, especially the intragranular carbides. SOL + AG IN625 displayed the precipitation of both equiaxial and elongated carbide, while SOL + AG IN625 + TiC highlighted mainly equiaxial-shaped particles with some precipitation of smaller particles inside grains.

Considering the carbides fraction (Figure 7e), the IN625 is subjected to a more drastic increment in the total concentration of carbides compared to the IN625 + TiC samples.

The SOL IN625 presented a concentration of carbides of 0.09% ± 0.04%, exhibiting sub-micrometric carbides, while the SOL + AG IN625 showed a concentration of carbides of 0.30% ± 0.20%, reporting average dimensions of 0.5 µm, with the presence of carbides with dimensions up to around 5 µm. SOL IN625 + TiC reported a total carbides concentration of 0.50% ± 0.10% with dimensions mainly ranging from 0.3 µm to 2 µm. Interestingly, the SOL + AG IN625 + TiC presented a total carbides concentration of 0.57% ± 0.20%, reporting a population of carbides ranging from 0.3 µm to 4 µm. This means that the prolonged thermal exposure produces a slight coarsening of the carbides, especially the intergranular carbides, which exhibit the largest determined sizes.

Overall, a more important carbide growth occurred in the IN625 alloy under prolonged heat treatment than in the composite, reporting a lower modification of the total carbide concentration. A possible speculation is that the carbon derived from the melted TiC particles triggers the formation of more carbides in the grain boundaries. Moreover, the solution-annealed treatment provoked the formation of other carbides, and the combined effect of the formed carbides and the TiC particles located in the grain boundaries and interdendritic areas hindered the recrystallization. As a consequence, a high quantity of carbon was used to form carbides, thus limiting the carbon for the formation of new carbides during subsequent thermal exposures.

**Figure 8:**
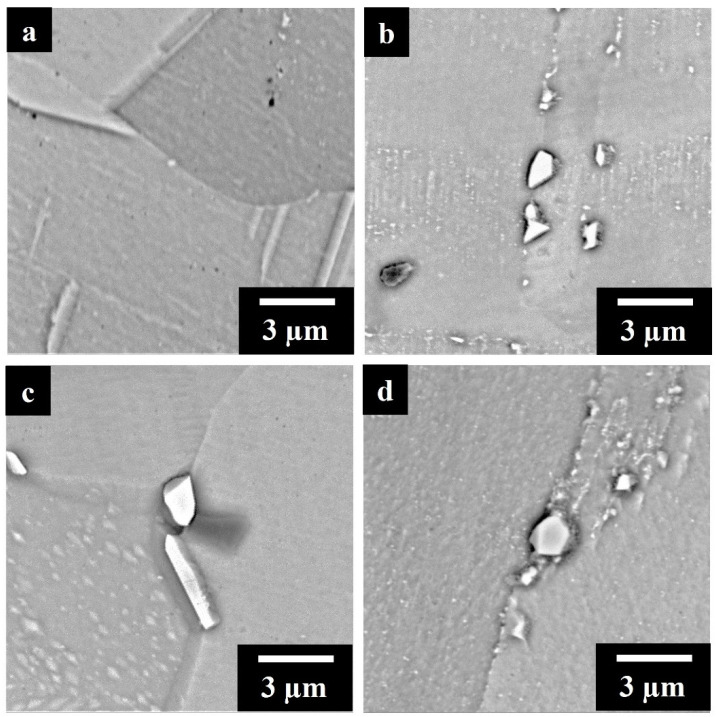
High magnification SEM images of etched (**a**) SOL IN625 (**b**) SOL IN625 + TiC (**c**) SOL + AG IN625 (**d**) SOL + AG IN625 + TiC.

**Figure A1:**
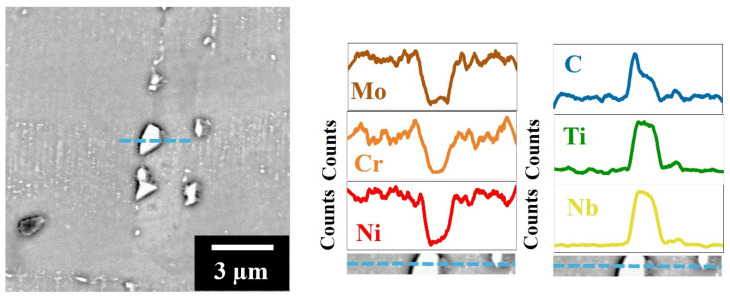
EDS analysis on SOL IN625 + TiC. The carbide can be identified as MC carbide.

Figure 8a–d shows the high-magnification SEM images of SOL and SOL + AG IN625, and IN625 + TiC. At higher magnification, SOL IN625 (Figure 8a) displayed the precipitation of sub-micrometric carbides. In previous work [40], these carbides in the SOL IN625 were detected as Nb, Ti-rich MC carbides by transmission electron microscopy (TEM) analysis. On the other hand, SOL IN625 + TiC observations (Figure 8b) depicted micrometric carbides along the grain boundaries, and the SEM + ESD analysis in Figure A1 in Appendix A pointed out the enrichment in Nb and Ti, compatible with the formation of Nb, Ti-rich MC carbides, based on the time–temperature–transformation (TTT) diagram of the IN625 alloy [16,17,25,41].

**Figure A2:**
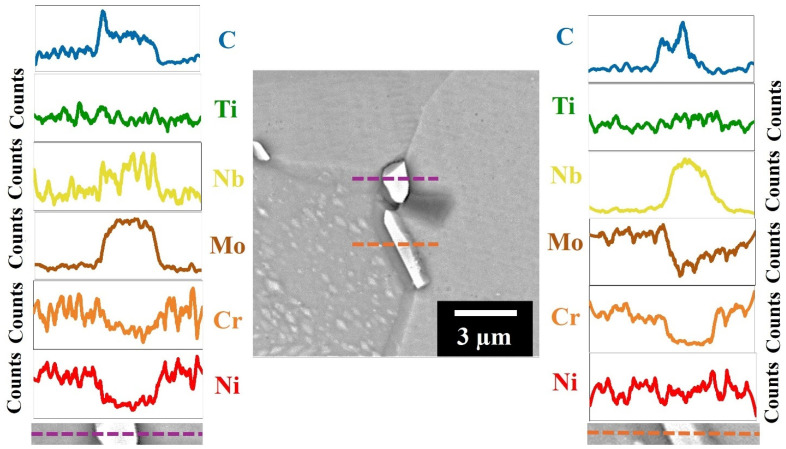
EDS analyses on SOL + AG IN625 carbides. The equiaxed carbide can be identified as M_6_C carbides while the elongated carbides can be attributed to MC carbides.

The fully treated SOL + AG IN625 (Figure 8c) samples showed a modified scenario compared to the SOL IN625 conditions. SEM images combined with EDS analysis in Figure A2 in Appendix A revealed carbides characterized by two different shapes. The large majority of the carbides presented equiaxed shapes with enrichment in Mo, Nb, and C, while the remaining carbides presented elongated shapes with enrichment in Nb and C. The first type can be associated with the formation of M_6_C carbides, while the second type can be identified as MC carbides.

**Figure A3:**
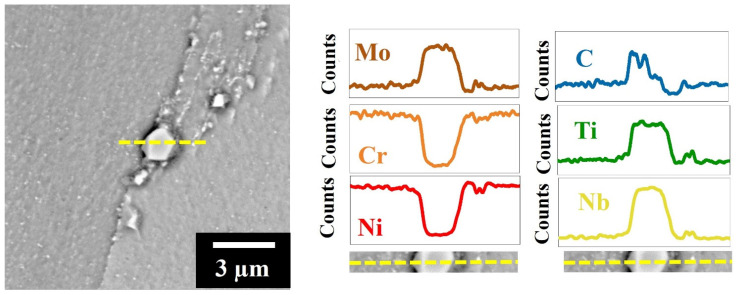
EDS analyses on SOL + AG IN625 + TiC carbide. The carbides can be attributed to the M_6_C carbides.

The SOL + AG IN625 + TiC images and EDS analysis (Figure 8d and Figure A3 in Appendix A) pointed out carbides along the grain boundaries mostly enriched with Nb, Ti, Mo, and C. The enrichment in Mo content suggested the identification as M_6_C. During prolonged heat treatments above 870 °C, MC carbide tends to decompose in M_6_C, characterized by an increment in Mo, in agreement with the identification of the carbides [16,30,42].

### 3.4. Microhardness Evaluation

**Figure 9:**
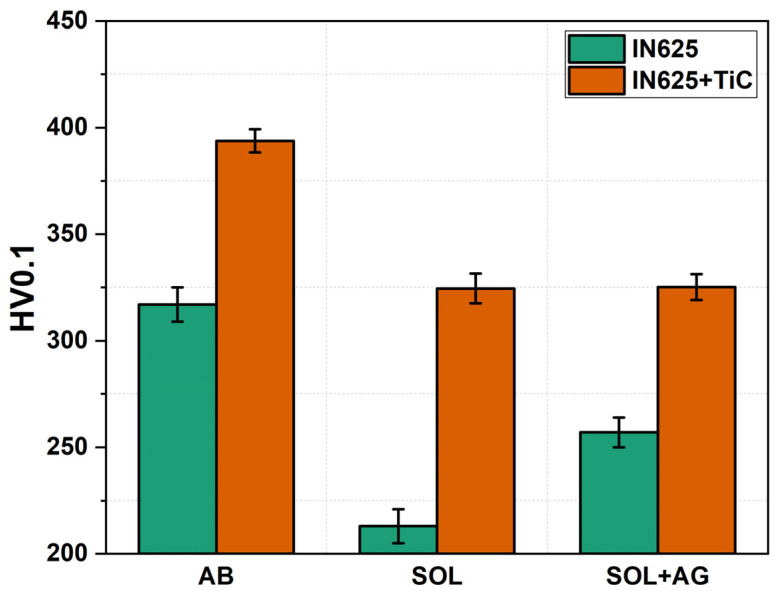
Vickers microhardness evolution of IN625 and IN625 + TiC in AB, SOL, and SOL + AG conditions.

Figure 9 shows the HV0.1 microhardness evolution of IN625 and IN625 + TiC through all the conditions investigated. The TiC addition increases the hardness value in all conditions acting as a hardening particle. Moreover, the availability of more Ti inside the gamma matrix increases the solid solution strengthening [13,26,27,39]. The AB IN625 + TiC reported an increment of 35% HV0.1 compared to the base alloy. Under heat treatment, IN625 reported softening in the solutioned conditions due to the total dissolution of the dendritic structures and the recrystallization, while the carbide precipitation during the soaking at 1000 °C promoted a slight increment in the hardness. Conversely, the heat-treated IN625 + TiC displayed a limited reduction in hardness value with respect to the AB composite. Compared to the base alloy, the IN625 + TiC samples reported significant improvements in SOL (from 213 HV0.1 to 359 HV0.1) and SOL + AG condition (from 257 HV0.1 to 363 HV0.1). Finally, the SOL + AG IN625 + TiC reported a hardness almost equal to the SOL conditions, thus highlighting its superior microstructure and hardness stability under prolonged thermal exposure.

Overall, the microhardness reduction after SOL and SOL + AG is limited for the IN625 + TiC with respect to the IN625 alloy. This could be important for applications at high temperatures since the material retains high hardness in the range of the AB IN625 material.

## 4. Conclusions

In the current work, the alloy IN625 and its composite reinforced with 2 wt.% of sub-micrometrical TiC were produced by LPBF and heat-treated to investigate their microstructure and hardness evolution. The as-built and heat-treated conditions were fully characterized by microstructural features and hardness measurements, and the main outcomes can be hereafter summarized:

- Despite the slight reduction in the flowability of composite powders, a VED of 99 J/mm^3^ allowed the production of dense IN625 + TiC samples with a maximum residual porosity of 0.15% comparable with the IN625 alloy. However, TiC segregations were still observable in the as-built composite and preferentially located along the melt pool boundaries.
- In the AB condition, both IN625 and IN625 + TiC displayed a highly stressed grain structure with melt pools, fine dendritic structures, and columnar grains elongated to the building direction. A small fraction of recrystallized grains was reported by GOS analysis with values of 20% and 28%, respectively, for the alloy and the composite. The differences could be associated with a beginning grain refinement effect induced by the presence of TiC.
- After SOL, IN625 underwent recrystallization, showing equiaxial grains. The fraction of recrystallized grains increased by 90%, and sub-micrometrical carbide precipitation was reported. The composite retained the AB features with columnar grains and a recrystallized grain fraction of 26%. At high magnification, the dendritic structures were still visible combined with micrometric and sub-micrometric carbides. The largest carbides were Nb- and Ti-rich MC carbides formed along the grain boundaries.
- After prolonged thermal exposures (SOL + AG condition), the IN625 and IN625 + TiC did not show the presence of remarkable grain growth. The SOL + AG IN625 displayed equiaxed grains with a fraction of recrystallized grains of 95%, while the composite revealed columnar grains still highly stressed with a recrystallization fraction steady at 25%. At high magnification, SOL + AG IN625 showed intense carbide precipitation and growth of micrometric carbides. On the other hand, the SOL + AG IN625 + TiC reported dimensions and concentrations of carbides similar to the SOL state. This observation suggested that a high quantity of carbon was already used to form carbides during the SOL heat treatment, and consequently, there is a limited growth of carbides during prolonged thermal exposure.
- The composite showed higher hardness in the AB, SOL, and SOL + AG conditions with respect to the IN625 alloy. It is interesting to note that the alloy is subjected to more hardness variations compared to the composite. In fact, the SOL IN625 suffered a strong softening due to the recrystallization, and then the carbide formation during SOL + AG increased the hardness. On the other hand, the IN625 + TiC retained elevated hardness values in the SOL and SOL + AG states due to a reduced microstructure variation.

This study proved the superior microstructure stability given by the addition of 2 wt.% of sub-micrometrical TiC to the IN625 matrix. The TiC particles promoted a more pronounced formation of MC carbides in the solution states, which inhibited the grain growth and recrystallization. After prolonged exposures, the carbides concentration remained similar to the solutioned state, thus keeping almost stable the microstructure and microhardness during prolonged exposure at high temperatures.

## Figures and Tables

**Table 1:** wt.% IN625 chemical composition evaluated with EDS and LECO combustion analysis.

Ni	Cr	Mo	Fe	Nb	Co	Si	Ti	Al	C
Bal.	22.3	8.4	0.9	3.6	0.5	0.2	0.3	0.2	0.020

**Table 2:** Flowability results of the IN625 and IN625 + TiC mixed powders.

Powder	Carney Flow Rate [s/150 g]
IN625 [27]	8.7 ± 0.1
IN625 1 wt.% TiC micrometric (1–5 µm) [26]	14.5 ± 0.5
IN625 2 wt.% TiC micrometric (1–5 µm) [26]	/
IN625 1 wt.% TiC sub-micrometric (mean size 200 nm) [27]	11.8 ± 0.3
IN625 2 wt.% TiC sub-micrometric (mean size 200 nm)	12.7 ± 0.1

## Data Availability

Data is contained within the article.

## Appendix A

Figure A1, Figure A2 and Figure A3 display the SEM/EDS analyses performed on carbides respectively for SOL IN625 + TiC, SOL + AG IN625, and SOL + AG IN625 + TiC.

SOL IN625 + TiC reported an enrichment of Nb, Ti, and C combined with Mo depletion associated with MC primary carbides. SOL + AG IN625 displayed two main types of carbides, the first enriched in Mo and C identified as secondary M_6_C carbides, while the second with Nb and C associated with MC carbides. The SOL + AG IN625 + TiC showed the large majority of carbides rich in Nb, Ti, Mo, and C thus allowing the identification as secondary M_6_C carbides.
